# A Homozygous Synonymous Variant Likely Cause of Severe Ciliopathy Phenotype

**DOI:** 10.3390/genes12060945

**Published:** 2021-06-21

**Authors:** Gulten Tuncel, Bahar Kaymakamzade, Yeliz Engindereli, Sehime G. Temel, Mahmut Cerkez Ergoren

**Affiliations:** 1 Rare Disease Research Group, DESAM Institue, Near East University, Nicosia 99138, Cyprus; gulten.tuncel@yahoo.com; 2 Department of Neurology, Faculty of Medicine, Near East University, Nicosia 99138, Cyprus; baharis83@yahoo.com; 3 Department of Child and Adolescent Psychiatry, Faculty of Medicine, Near East University, Nicosia 99138, Cyprus; Yeliz.engindereli@neu.edu.tr; 4 Department of Medical Genetics, Faculty of Medicine, Bursa Uludag University, Bursa 16059, Turkey; sehime@uludag.edu.tr; 5 Department of Histology and Embryology, Faculty of Medicine, Bursa Uludag University, Bursa 16059, Turkey; 6 Department of Translational Medicine, Institute of Health Sciences, Bursa Uludag University, Bursa 16059, Turkey; 7 Department of Medical Genetics, Faculty of Medicine, Near East University, Nicosia 99138, Cyprus

**Keywords:** Joubert syndrome, *AHI1*, *CC2D1A*, ciliopathy

## Abstract

Joubert syndrome (OMIM #213300) is a rare neurodevelopmental disease characterized by abnormal breathing patterns, intellectual impairment, ocular findings, renal cysts, and hepatic fibrosis. It is classified as a ciliopathy disease, where cilia function or structure in various organs are affected. Here, we report a 17-year-old male whose main clinical findings are oculomotor apraxia and truncal ataxia. Magnetic resonance imaging revealed the characteristic molar tooth sign of Joubert syndrome. He also has obsessive–compulsive disorder concomitantly, which is not a known feature of Joubert syndrome. Molecular genetic analysis revealed a homozygous c.2106G>A (p.(Thr702=)) variation in the Abelson helper integration 1 (*AHI1*) gene and another homozygous c.1739C>T (p.Thr580Ile) variation in the coiled-coil and C2 domain-containing protein 1A (*CC2D1A*) gene. Even though certain *AHI1* variations were previously associated with Joubert syndrome (JS), c.2106G>A (p.(Thr702=)) was only reported in one patient in trans with another known pathogenic JS variant. The *CC2D1A* c.1739C>T (p.Thr580Ile) variation, on the other hand, has been reported to cause autosomal recessive nonsyndromic mental retardation, but there are conflicting interpretations about its pathogenicity. Overall, to our knowledge, this is the first patient representing a severe ciliopathy phenotype caused by a homozygous synonymous *AHI1* variation. Further investigations should be performed to determine any involvement of the *CC2D1A* gene in ciliopathy phenotypes such as Joubert syndrome.

## 1. Introduction

Joubert syndrome (JS) (OMIM #213300) is a rare disease with an estimated prevalence of 1:100,000 live births worldwide and is inherited in an autosomal recessive or X-linked manner [1]. JS can affect many body parts, but it is primarily a disorder of brain development. It is characterized by a malformed brain stem (molar tooth appearance), which normally controls breathing and swallowing reflexes. Additionally, absence or underdevelopment of the cerebellar vermis that regulates balance and coordination is also observed in JS. As these regions are affected, patients suffer from ataxia, oculomotor apraxia, abnormal breathing patterns (hyperpnea), and sleep apnea starting from early childhood. Additionally, muscular hypotonia, intellectual disability, retinal dystrophy, polydactyly, and renal disease are also common symptoms that can be associated with JS in patients.

To our knowledge, there are around 35 genes involved in JS, overall accounting for 60% of all cases [2]. These genes include *AHI1*, *TMEM216*, *NPHP1*, *CEP290*, *TMEM67*, *RPGR1P1L*, *ARL13B*, *CC2D2A*, *CXORF5*, *TTC21B*, *KIF7*, *TCTN1*, *TMEM237*, *CEP41*, *TMEM138*, *C5ORF42*, *TCTN3*, *ZNF423*, *TMEM231*, *CSPP1*, *PDE6D*, *KIAA0586*, *TCTN2*, *CEP104*, *KIAA0556*, *B9D1*, *MKS1*, *TMEM107*, and *ARMC9* [1,2,3,4,5,6,7,8,9,10,11,12,13,14,15,16,17,18,19,20,21,22,23,24]. The most important common properties of these genes are that they code for proteins involved in the structures of cilia and related basal body or transition zone, and pathogenic variations in these genes result in abnormalities in the structure and function of cilia and related structures. Functional abnormalities in cilia and structures forming the cilia result in around 15 different diseases, including JS, and this group of diseases are referred as “ciliopathies” [25]. Orofaciodigital syndrome, Meckel–Gruber syndrome, Alström syndrome, and Bardet–Biedl syndrome are other examples of ciliopathy diseases.

In the last two decades, discovery of rare variants and molecular mechanisms in disease resulted in an increase in the number of research studies about cilia and related diseases. Applying advanced Deoxyribonucleic acid (DNA) sequencing techniques is enhancing our understanding of the genotype–phenotype relationships in diseases with extreme genetic heterogeneity such as JS. In this report, we present a patient carrying homozygous variations in *AHI1* (NM_001134831.2) and *CC2D1A* (NM_017721.5) genes, who was diagnosed with JS coexistent with obsessive–compulsive disorder.

## 2. Materials and Methods

### 2.1. Subject

**Figure 1:**
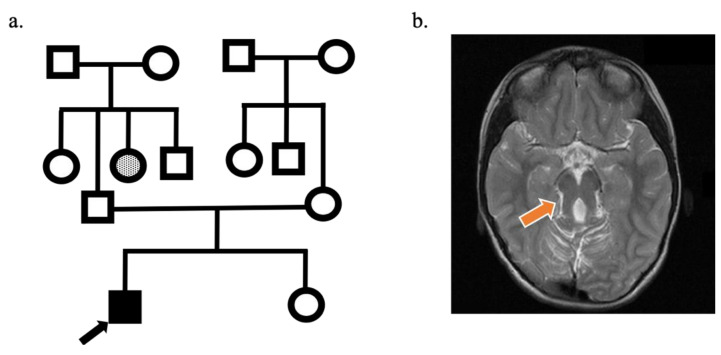
(**a**) Family pedigree of the proband is shown. Proband is the first child of nonconsanguineous parents. Parents and younger sister are not affected. (**b**) Molar tooth image observed in the brain magnetic resonance imaging of the proband is indicated by the arrow.

A 17-year-old male patient was admitted to the Near East University Medical Faculty Hospital Neurology outpatient clinic with the chief complaints of imbalance and eye movement abnormalities since birth. He was the first child of nonconsanguineous healthy parents from Turkish Cypriot heritage. He was born by normal vaginal delivery at 3250 grams. Family pedigree is presented in Figure 1a.

### 2.2. Molecular Genetic Analysis

Whole exome sequencing (WES) was performed to confirm diagnosis. Genetic analysis was performed after obtaining informed consent from the parents. DNA isolated from peripheral blood samples was used in sequencing experiments. An Illumina TruSight One Sequencing Panel and MiSeq sequencer (Illumina, San Diego, CA, USA) was used for targeted resequencing of the DNA sample following in-solution target enrichment according to the manufacturer’s protocols. An in-house pipeline was used for sequence alignment to the reference genome, variant calling, annotation, and filtering in order to eliminate benign single nucleotide polymorphisms with allele frequencies ≤0.03. All rare homozygous variants with a minor allele frequency (MAF) <1% in the 1000 genome phase III and Exome Aggregation Consortium (ExAC) database and the public database of single nucleotide variants (dbSNP) were screened. The Human Gene Mutation Professional Database (HGMD) was used to screen previously reported clinical significance of the detected variants.

Genetic variations were confirmed by polymerase chain reaction (PCR) coupled with direct sequencing of target regions on a CEQ8800 sequencer (Beckman Coulter) according to the manufacturer’s protocols.

## 3. Results

### 3.1. Neurological and Neuropsychiatric Findings

The patient had delayed motor and cognitive milestones in his past medical history. His mother described symptoms consistent with neonatal episodic hyperpnea and hypotonia for the early childhood period. On neurological examination, he had inability to initiate voluntary saccades in a head-fixed position, while saccades can be initiated by the vestibulo-ocular reflex, which is suggestive of oculomotor apraxia (Video S1) and truncal ataxia (Video S1). Brain magnetic resonance imaging (MRI) revealed that cerebellar vermis hypoplasia, mesencephalon, and superior cerebellar peduncles were seen to constitute the typical molar tooth sign of Joubert syndrome (JS) (Figure 1b).

The patient was also evaluated by the child and adolescent psychiatry department because of psychiatric symptoms, which started 2 years ago and was characterized by repetitive questions, extreme anxiety and anger when not answered by his mother, and worrying about getting sick. Neuropsychological testing was performed after clinical interview using the Wechsler Intelligence Scale for Children (WISC), revised edition, and he demonstrated moderate intellectual disability and was diagnosed with obsessive–compulsive disorder (OCD).

Retinal examination and abdominal ultrasonography were performed, and conditions outside of the nervous system related to JS, including retinal dystrophy, ocular coloboma, cystic renal disease, and hepatic fibrosis were excluded.

### 3.2. Molecular Genetic Findings

**Figure 2:**
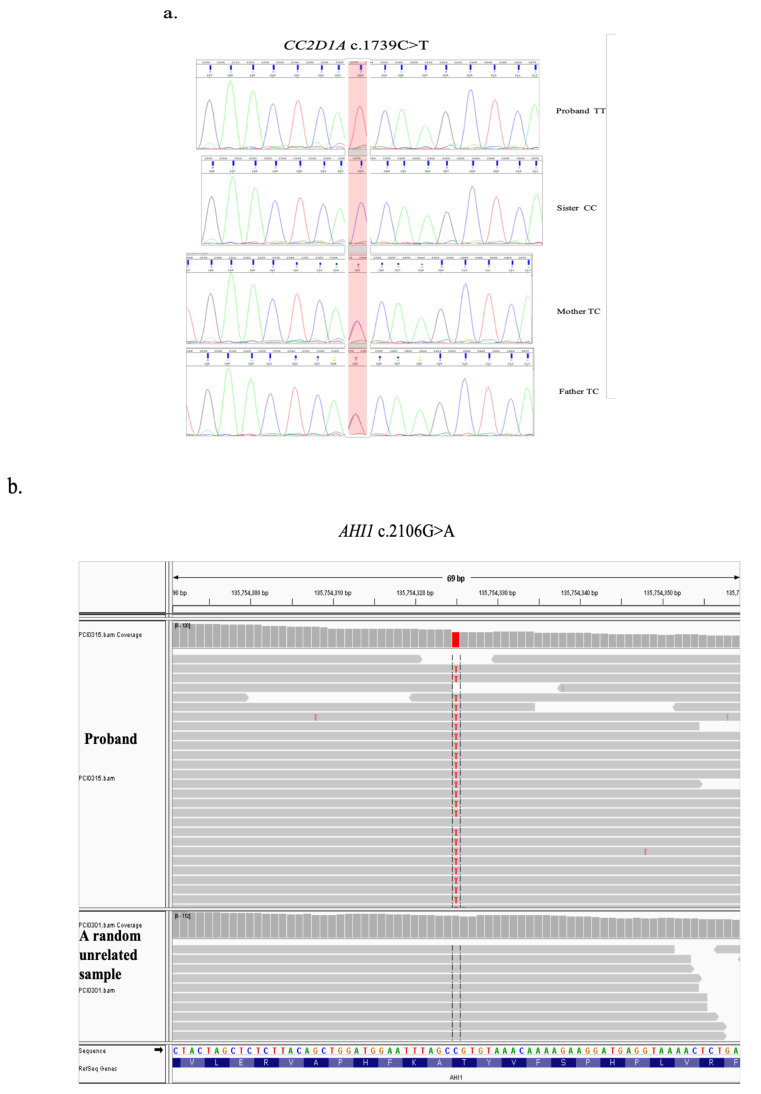
(**a**) Sanger sequencing results of the *CC2D1A* c.1739C>T variation in the proband (homozygous T), sister (homozygous C), and parents (heterozygous). (**b**) Whole genome sequencing results of the *AHI1* c.2106G>A variant of the proband compared with random unrelated samples.

Whole exome sequencing was performed to determine genetic variants underlying the phenotype observed in our patient. Results revealed a homozygous c.2106G>A (p.(Thr702=)) variation in the Abelson helper integration 1 (*AHI1*) gene (OMIM# 608894) and another homozygous c.1739C>T (p.Thr580Ile) variation in the coiled-coil and C2 domain-containing protein 1A (*CC2D1A*) gene (OMIM# 610055) in the proband inherited from heterozygous parents (Figure 2).

*AHI1* c.2106G>A (p.(Thr702=)) is a silent variation at codon 702 and was previously reported in heterozygous state in a JS patient, in trans with another known pathogenic JS variant in ClinVar and HGMD [26].

*CC2D1A* c.1739C>T (p.Thr580Ile) is a missense variant causing substitution of threonine with isoleucine at position 580 of the CC2D1A amino acid sequence. The variant was previously reported to be associated with Smith–Magenis syndrome-like and mental retardation autosomal recessive 3 disorder in ClinVar and HGMD. There are conflicting interpretations of pathogenicity in ClinVar [27].

## 4. Discussion

Joubert syndrome (JS) is a rare genetic disorder, and the term is used for individuals who fulfill only the diagnostic criteria of developmental delay, abnormal ocular movements, radiological evidence of molar tooth sign, and cerebellar vermis changes [28]. The term “JS and related disorders” (JSRDs) is used to refer to a group of conditions presenting the pathognomonic features of JS associated with the involvement of other organs and systems [29]. JSRDs are classified according to genotype–phenotype correlation. These include pure JS, JS with ocular defect, JS with renal defect, JS with oculorenal defects, JS with hepatic defect, and JS with orofaciodigital defects [30]. Our patient was diagnosed with pure JS as he has the characteristic molar tooth sign on MRI, moderate intellectual disability, truncal ataxia, and hypotonia symptoms associated with the disease phenotype. However, severe obsessive–compulsive disorder observed in our patient is not a known feature of JS.

JS is classified as a ciliopathy disorder as the genes associated with the disease are all involved in ciliogenesis or cilia function. Pathogenic variations of these genes cause defective cilia, resulting in phenotypes with varying types and severities. It was reported that the type of the two recessive variants can determine the severity of the disease. Nonsense mutations are classified as strong mutations, whereas missense and synonymous mutations are weak. Presence of two strong mutations results in more severe early-onset developmental disorders with a broad-range organ involvement as in Meckel syndrome. Presence of at least one weak mutation, however, causes a milder late-onset degenerative disorder, such as a mild form of JS [31]. The synonymous AHI1 variant detected in our patient does not seem to follow this rule.

Abelson helper integration 1 (*AHI1*) is one of the genes associated with JS, which is expressed in embryonic brain development and is required for both cerebellar and cortical development in humans. Pathogenic *AHI1* variations are well defined in patients with JS and related disorders [32]. The *AHI1* c.2106G>A (rs1276908141) variant detected in our patient is a silent change in codon 702, with no effect on the amino acid sequence. No homozygous individuals were reported in gnomAD. The global allele frequency of the variant is shown as 0.0004%, reported only in one person in a heterozygous state in the non-Finnish European population. According to ClinVar, this variant was detected in trans with a pathogenic *AHI1* (p.Pro560Thrfs*) variant in an individual with JS, suggesting that c.2106G>A (rs1276908141) may contribute to the disease phenotype in this patient; therefore, it was classified as likely pathogenic [33]. To our knowledge, our patient is the first case reported in the literature carrying the homozygous rs1276908141 variant with clinical features consistent with JS.

In the ClinVar database, it was noted that prediction algorithms predict that the variation may create or strengthen a new splice site. However, when we utilized the NNSplice prediction tool, a neural network modeling program, no changes in acceptor or donor site predictions were shown (Supplementary File S1).

Apart from the *AHI1* variant, a homozygous variation in coiled-coil and C2 domain-containing protein 1A (*CC2D1A*), also known as Freud-1, Aki1, and MRT3, was also detected in our patient. CC2D1A is known to regulate the expression of 5-hydroxytryptamine receptor 1A (5-HTR1A) in neuronal cells and also activate nuclear factor κ enhancer binding protein (NF-κB) through the canonical IκB kinase complex (IKK) pathway [34,35]. When in the centrosomes, it is involved in the regulation of spindle pole localization of the cohesin subunit SCC1/RAD21 and mediate centriole cohesion during mitosis [36]. Variations in the gene were previously associated with autosomal recessive nonsyndromic mental retardation [37].

Although there are conflicting interpretations of pathogenicity in the ClinVar database, the c.1739C>T (rs202057391) variant detected in our patient was previously reported to be associated with Smith–Magenis syndrome-like disorder and was classified as pathogenic in one publication [38]. Additionally, the variant was classified as “damaging” according to the Fathmm-MKL (Functional Analysis through Hidden Markov Models) prediction software [39]. Cellular and molecular mechanisms by which the *CC2D1A* may be involved in these neuropsychiatric-related behaviors are not clear. A recent study by Ma et al. revealed that loss of function of *CC2D1A* in the zebrafish model results in ciliary dysfunction and left–right patterning, suggesting that the gene may be associated with ciliary dysfunction [40]. Furthermore, preliminary results using *Xenopus tropicalis* as a model organism suggests that the protein is involved in cilia structure and function as loss of epidermal flow and a reduced number of multiciliated cells were observed in *CC2D1A*-knockout tadpole skin [41]. However, variant-specific models are needed to analyze the precise effect of variations.

On the contrary, the variant was classified as benign or likely benign in other ClinVar submissions. Additionally, there are five homozygotes reported in gnomAD for this variant. Calculated global allele frequencies are 0.5% and 0.6% in the European population, which are considered high for a potential pathogenic variant.

In light of this information, as there are no functional data supporting a loss-of-function effect of the c.1739C>T (rs202057391) *CC2D1A* variant and there are five healthy homozygotes, it is unlikely that the variation had a deleterious effect alone. However, as recent data suggest that the CC2D1A protein has cilia-related functions, we think that the presence of this homozygous missense variation may enhance the effect of the homozygous synonymous *AHI1* variant. It is known that the involvement of mutations in other modifier genes or the combined effect of two or more recessive genes with heterozygous mutations (true oligogenicity) is important in the determination of genotype–phenotype correlations of the ciliopathy phenotype [31,42].

In conclusion, we report a patient with a Joubert syndrome phenotype caused by the homozygous c.1739C>T (rs202057391) *AHI1* variant for first time in the literature. Another homozygous missense variant in the *CC2D1A* gene, which was recently suggested to be involved in cilia function, is likely to be a benign variant when present alone; however, the variation might be causing protein interactions or regulatory changes that may enhance the ciliopathy phenotype in the presence of another pathogenic variation.

Further functional analysis of the gene–gene and gene–phenotype interactions will help us understand the molecular mechanisms involved in the disease phenotype, and also is of great importance to inform future patient-specific treatment options for affected individuals.

## Supplementary Materials

The following are available online at https://www.mdpi.com/article/10.3390/genes12060945/s1: Video S1: Shows the neurological examination findings of the patient. (**a**) The patient cannot initiate saccadic eye movements in order to look at a point in a desired direction and has to turn his head in order to compensate for the lack of eye movement initiation. (**b**) The wide-based ataxic gait is seen. Supplementary File S1: Splice site predictions.
