# Supplementary material for: A Homozygous Synonymous Variant Likely Cause of Severe Ciliopathy Phenotype
Source: Genes (Basel). 2021 Jun 21;12(6):945. doi: 10.3390/genes12060945 (PMC8234327; doi:10.3390/genes12060945)
Supplement: Supplementary file 1 [file genes-12-00945-s001.zip › genes-1226018-supplementary.pdf]

Splice site predictions for 2 sequences with donor score cutoff 0.40, acceptor score cutoff 0.40 (exon/intron boundary shown in larger font):

Donor site predictions for NM_001134831.2 :

Start	End	Score	Exon Intron
596	610	0.99	tgatgatgtaagtgc
861	875	0.62	cagaaaagtgagaag
1101	1115	0.42	gaaacaagtacattg
1448	1462	0.89	cccaatggtaaaaat
1942	1956	0.88	caacactgtacgtaa
1952	1966	0.79	cgtaactgtaagagg
2025	2039	0.97	gaaaaaggtaaacca
3317	3331	0.50	cacagaggtgatacg
3500	3514	0.90	ccatcaggtagatac
4486	4500	0.89	acctaaggtcaggag
4821	4835	0.60	catataggtaacttc
5341	5355	0.69	aaaattggtaaacat
5497	5511	0.59	gtgtcaggtacagct
5866	5880	0.98	tgacaaagtaagact

Acceptor site predictions for NM_001134831.2 :

Start	End	Score	Intron Exon
3	43	0.86	agagccgcggcttgctttcaggacacgggtcgctggcgggt
140	180	0.68	gttctgcgtacccctctccagtgtcaacctggggctgaatc
318	358	0.57	gtcaatgctttgcattctcagtcctctgcataaagctgaga
1003	1043	0.97	agttgacttactttccctcagatactttattccatgatgac
1162	1202	0.90	cagttagatcagtttcttcagattctcatcaagatgatgaa
2001	2041	0.77	cgctctatgatggctcttcaggaggaaaaaggtaaaccagt
2605	2645	0.67	cactttgttttgatactgaaggtcatcatatgtattcagga
3114	3154	0.41	gatttccatgttgcccagcaggaggctgaaatgttcaaacg
3225	3265	0.95	ccccatcaaggctcttttcagattgatgaatttgtccacac
4005	4045	0.80	ccaaatggaatttctcttcagagttcagaattttcagatac
4793	4833	0.58	taaaattttgtgtttcttaagatcaaatcatataggtaact
5002	5042	0.80	tttattttttctaagttccagaataatagtgtcattattat
5590	5630	0.94	aaaatatctgtttctctgcagtttcagctaataattttgag

Donor site predictions for NM_001134831.2(AHI1):c.2106G>A :

Start	End	Score	Exon Intron
596	610	0.99	tgatgatgtaagtgc
861	875	0.62	cagaaaagtgagaag

1101	1115	0.42	gaaacaagtacattg
1448	1462	0.89	cccaatggtaaaaat
1942	1956	0.88	caacactgtacgtaa
1952	1966	0.79	cgtaactgtaagagg
2025	2039	0.97	gaaaaaggtaaacca
3317	3331	0.50	cacagaggtgatacg
3500	3514	0.90	ccatcaggtagatac
4486	4500	0.89	acctaaggtcaggag
4821	4835	0.60	catataggtaacttc
5341	5355	0.69	aaaattggtaaacat
5497	5511	0.59	gtgtcaggtacagct
5866	5880	0.98	tgacaaagtaagact

Acceptor site predictions for NM_001134831.2(AHI1):c.2106G>A :

Start	End	Score	Intron Exon
3	43	0.86	agagccgcggcttgctttcaggacacgggtcgctggcgggt
140	180	0.68	gttctgcgtacccctctccagtgtcaacctggggctgaatc
318	358	0.57	gtcaatgctttgcattctcagtcctctgcataaagctgaga
1003	1043	0.97	agttgacttactttccctcagatactttattccatgatgac
1162	1202	0.90	cagttagatcagtttcttcagattctcatcaagatgatgaa
2001	2041	0.77	cgctctatgatggctcttcaggaggaaaaaggtaaaccagt
2605	2645	0.67	cactttgttttgatactgaaggtcatcatatgtattcagga
3114	3154	0.41	gatttccatgttgcccagcaggaggctgaaatgttcaaacg
3225	3265	0.95	ccccatcaaggctcttttcagattgatgaatttgtccacac
4005	4045	0.80	ccaaatggaatttctcttcagagttcagaattttcagatac
4793	4833	0.58	taaaattttgtgtttcttaagatcaaatcatataggtaact
5002	5042	0.80	tttatttttttctaagttccagaataatagtgtcattattat
5590	5630	0.94	aaaatatctgtttctctgcagtttcagctaataattttgag
